# Correction: Back to the light, coevolution between vision and olfaction in the “Dark-flies” (*Drosophila melanogaster*)

**DOI:** 10.1371/journal.pone.0243035

**Published:** 2020-11-30

**Authors:** Ismet Özer, Thomas Carle

The images for all the figures are incorrect. The figure captions are correct and appear in the correct order. Please see the correct Figs 1–5 here.

**Fig 1:**
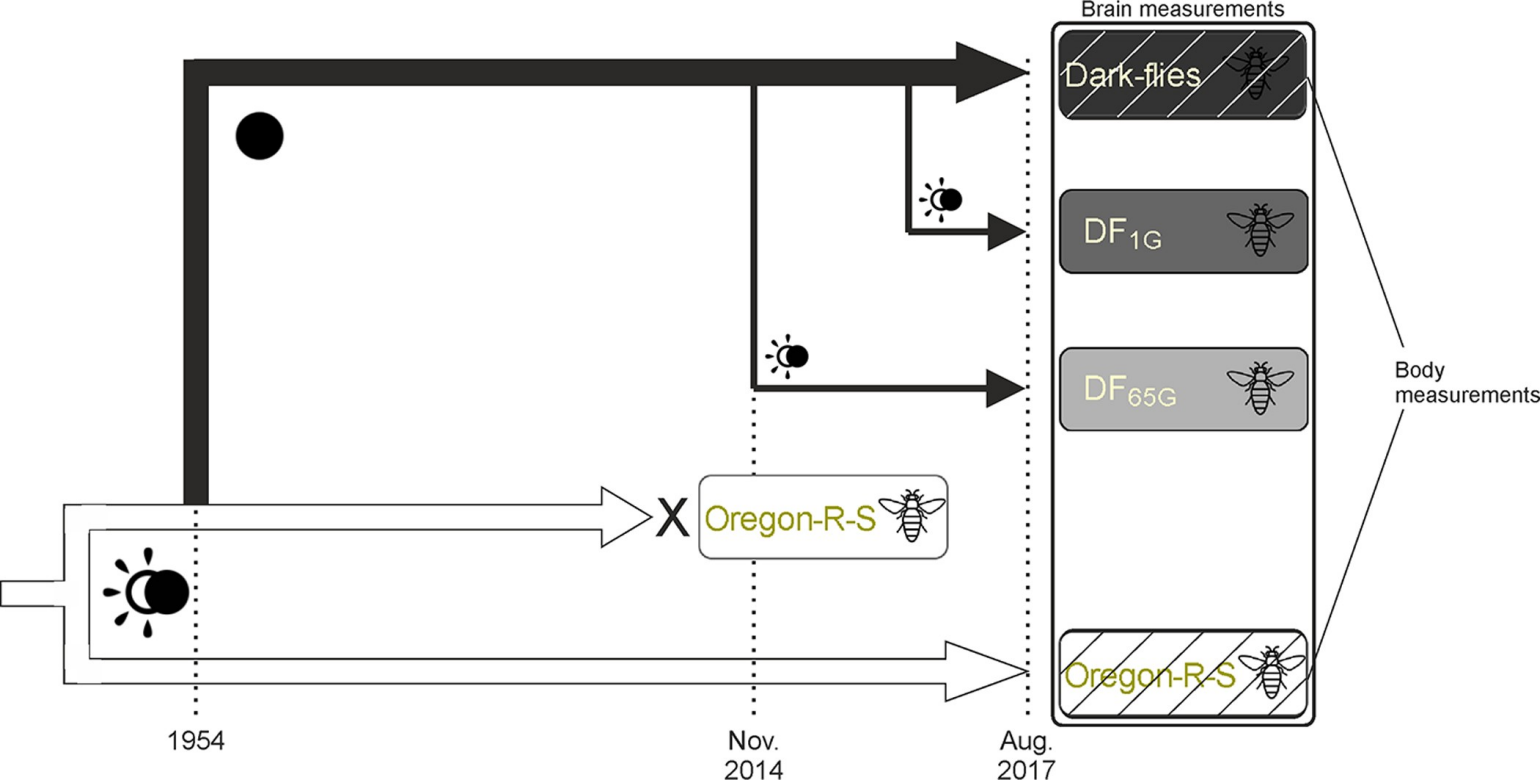
Representation of the experimental design and timeline.

**Fig 2:**
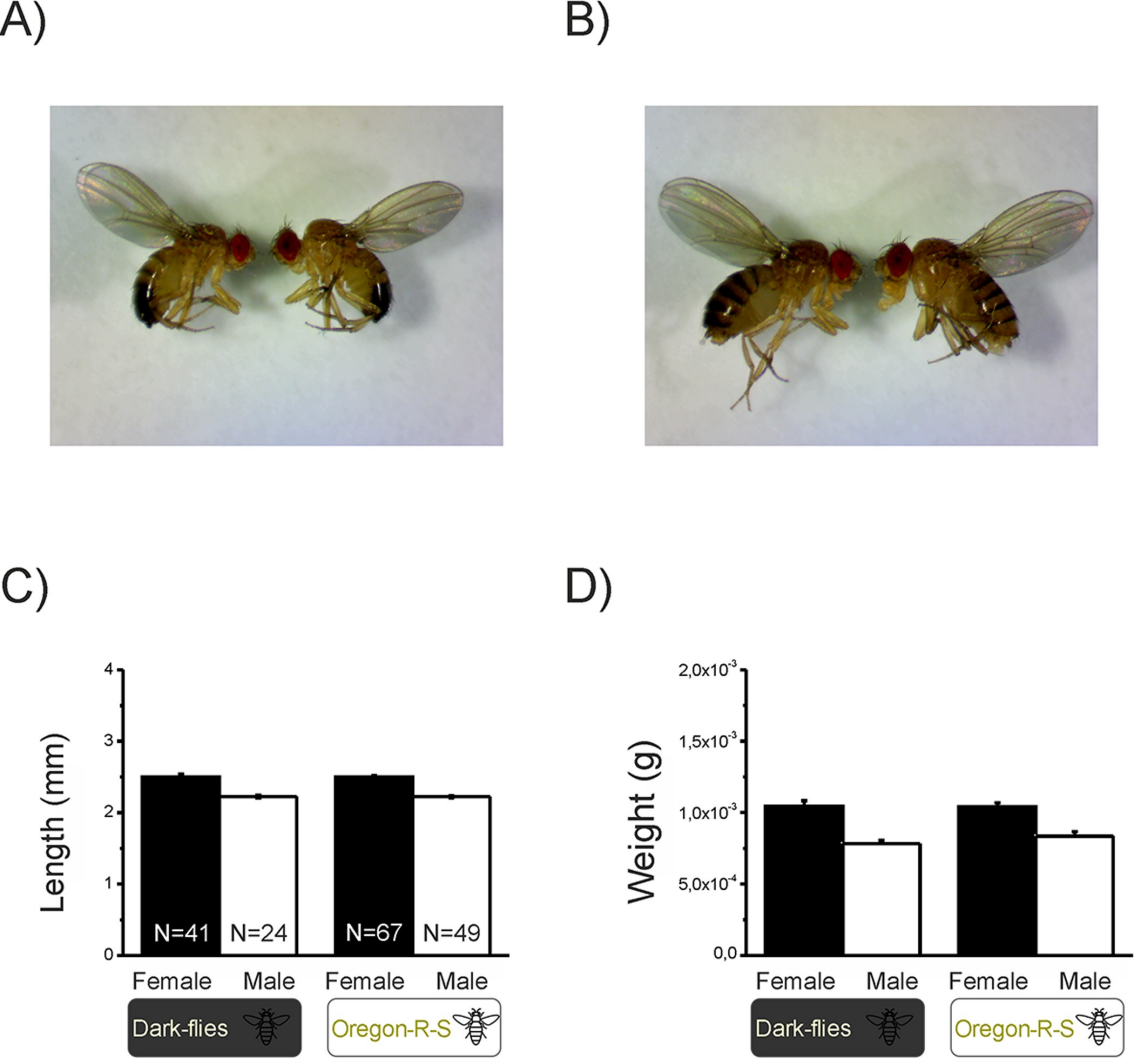
**Images of male (A) and female (B) flies from each strain.** In each image, Dark-flies are on the left, and Oregon flies on the right. The mean (+SEM) length (C) and mass (D) of male and female Oregon flies and Dark-flies.

**Fig 3:**
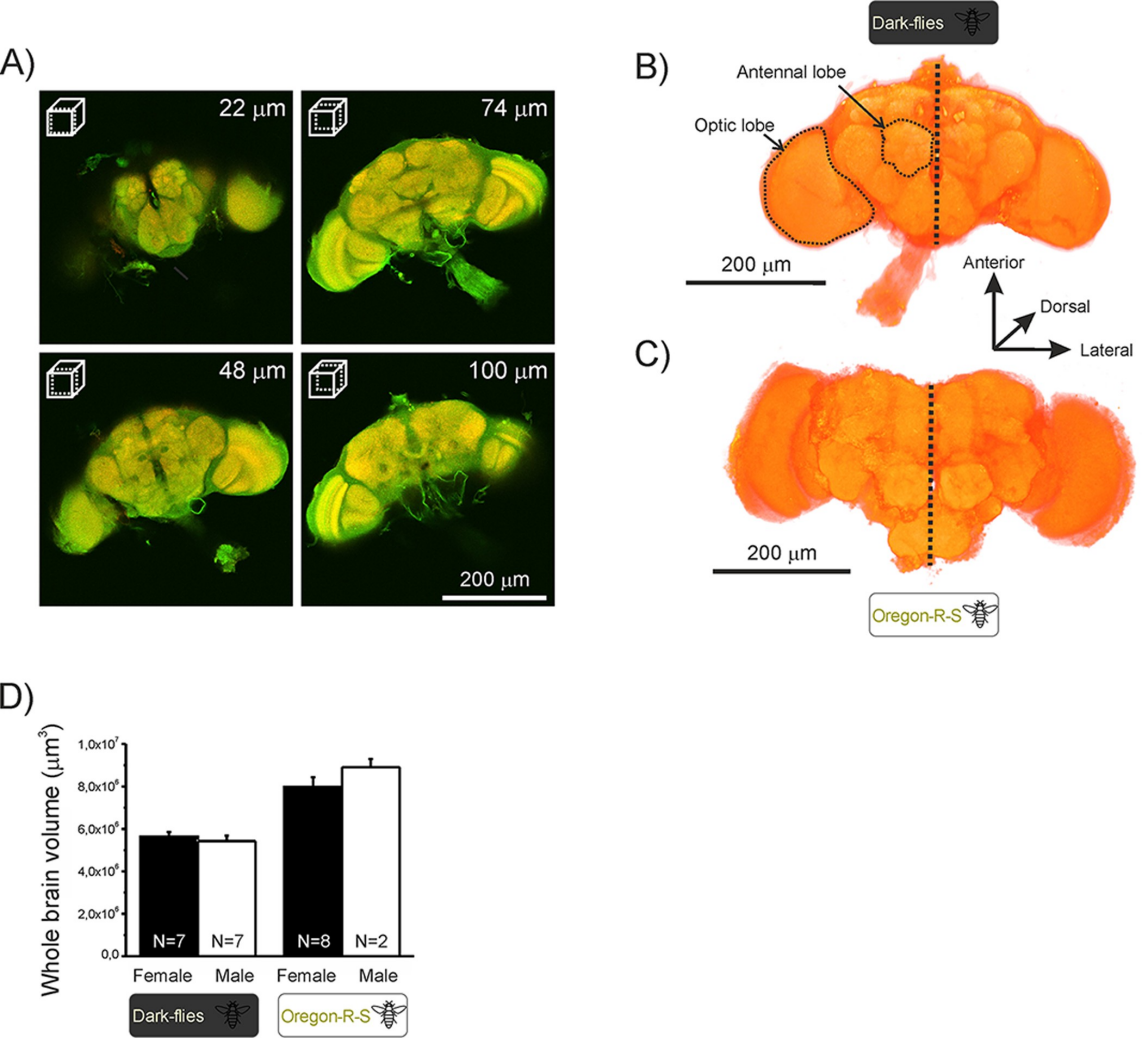
Flies’ brains. Brain images from a brain of a male Dark-fly showing a series of optical sections from ventral (top left) to dorsal (bottom right) side following a neural axis (A). A 3D representation of a brain of a male Dark-fly (B) and a male Oregon fly (C). The scales and depth location are presented in the figure. The mean (+SEM) size of whole brain (including OLs and ALs) (D) in males and females Oregon and Dark-flies.

**Fig 4:**
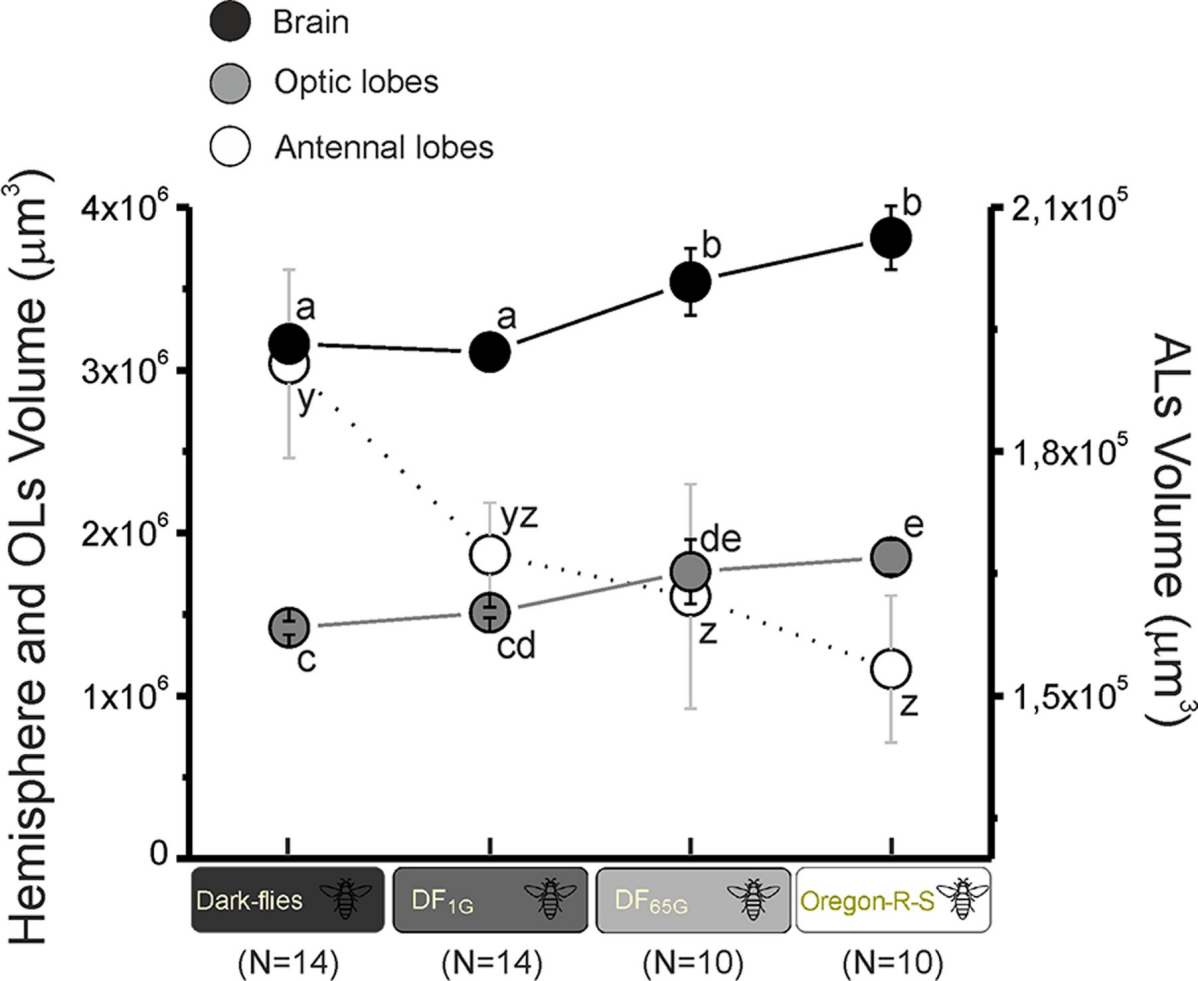
Hemisphere, OLs and ALs absolute sizes. Absolute volumes ± SEMs of hemisphere (black), OLs (grey) and ALs (white) in Dark-flies, DF_1G_, DF_65G_ and Oregon flies. Statistics are represented with letters associated to dots, and significant differences (p<0.05) are represented with different letters.

**Fig 5:**
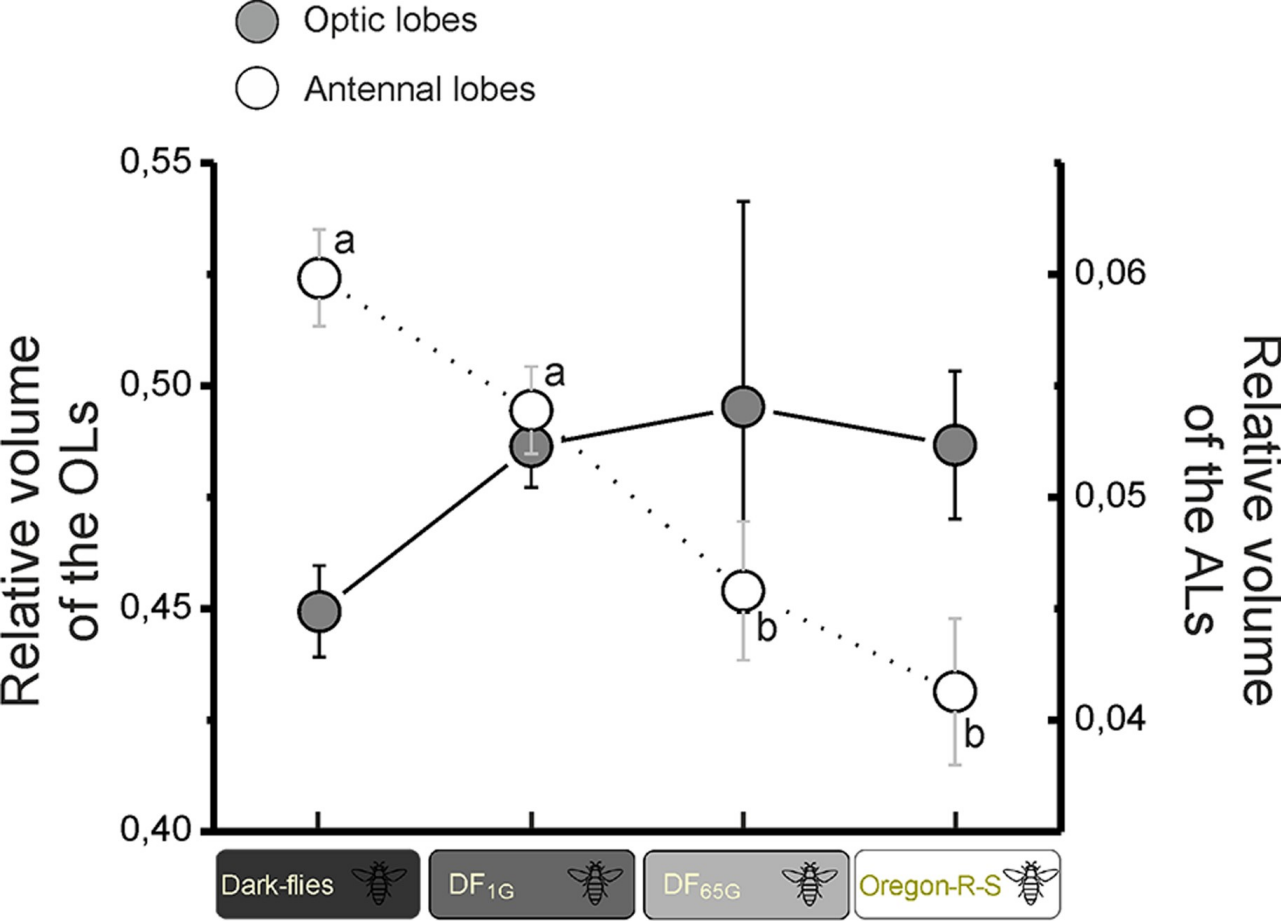
OLs and ALs relative sizes. Mean values of the relative sizes ± SEMs of OLs (grey) and ALs (white) in Dark-flies, DF_1G_, DF_65G_ and Oregon flies. The number of individuals used is shown in Fig 4. Statistics are represented with letters associated to dots, and significant differences (p<0.05) are represented with different letters.
