# Supplementary figures and images for: Back to the light, coevolution between vision and olfaction in the “Dark-flies” (Drosophila melanogaster)
Source: PLoS One. 2020 Feb 11;15(2):e0228939. doi: 10.1371/journal.pone.0228939 (PMC7012446; doi:10.1371/journal.pone.0228939)

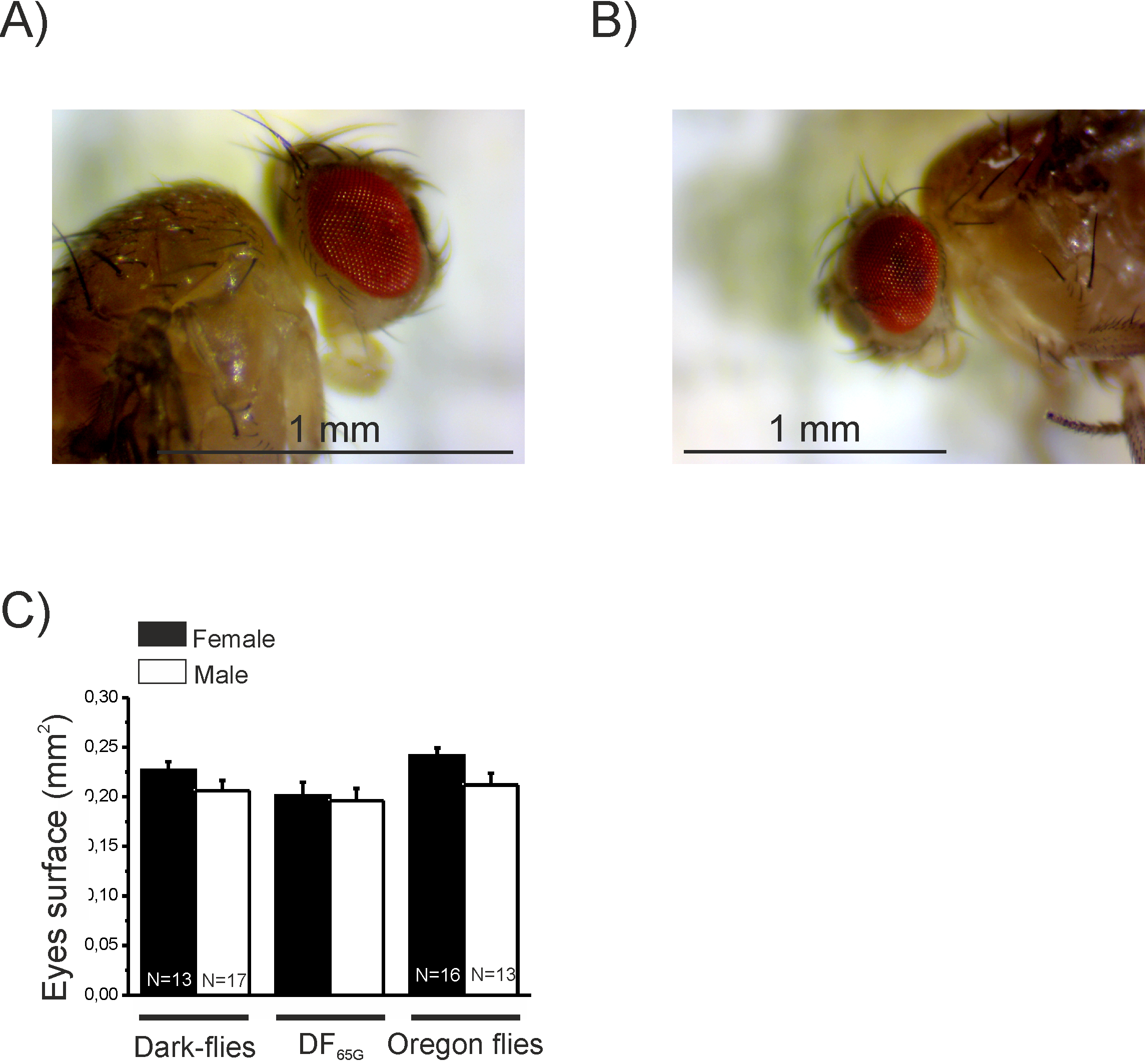

Supplement: S1 Fig — Images of the eyes of Dark-flies (A) and Oregon flies (B) in females (top) and males (bottom). The mean (+SEM) surface of a single eye (c) of male and female Dark-flies and Oregon flies. (TIF) [file pone.0228939.s001.tif]
