# Supplementary material for: Back to the light, coevolution between vision and olfaction in the “Dark-flies” (Drosophila melanogaster)
Source: PLoS One. 2020 Feb 11;15(2):e0228939. doi: 10.1371/journal.pone.0228939 (PMC7012446; doi:10.1371/journal.pone.0228939)
Supplement: S1 File — (PDF) [file pone.0228939.s002.pdf]

Methods

Eyes measurements

At 4 days old, Oregon flies and Dark-flies that had been kept under their standard rearing conditions were collected to measure the size of single eye (Oregon: 13 males, 16 females; Dark-flies: 17 males, 13 females; Supplemental Fig. 1). The flies were anesthetised by putting their tubes into ice. Photographs of the flies were taken against a piece of graph paper (for scale) using a camera fixed on a microscope (Brunel eyecam plus fixed to a BMDZ Brunel Microscopes Ltd). The eye surface of each fly was measured using GIMP 2 (version 2.8.22).

Results

Eye size

Dark-flies and Oregon flies also did not differ in their eye size (Fig. 2C). Whilst females had slightly bigger eyes than males (GLM; χ^2_1=20.41, P<0.001; Fig. 2C), we did not find any effect of strain on eye area (χ^2_1=0.002, P>0.05), nor interaction between sex and strain (χ^2_1=0.73, P>0.05).
