# Supplementary material for: Back to the light, coevolution between vision and olfaction in the “Dark-flies” (Drosophila melanogaster)
Source: PLoS One. 2020 Feb 11;15(2):e0228939. doi: 10.1371/journal.pone.0228939 (PMC7012446; doi:10.1371/journal.pone.0228939)
Supplement: Attachment — Submitted filename: Review_BackToLight.doc [file pone.0228939.s003.doc]

Review

Overall the paper is interesting, the methods, results and the conclusions seem generally credible. However, the manuscript needs major revisions in terms of clarity. In the review below only several specific cases where the revisions is necessary are stated, however, the manuscript probably needs major revisions way beyond these examples. In particular the hypothesis and predictions should be clearly stated. The Figures and the Results should address the predictions clearly and any discrepancies between the predictions and the results should be pointed out and thoroughly discussed. The justifications for the control groups used should also be made more clear.

This reviewer recommends the manuscript for publication based on intriguing, indeed fascinating, question of how development and evolution of sensory systems can be affected by depriving flies of one sensory modality (vision) across many generations, in a controlled environment. The reviewer is confident that the authors will be able to present their current results and conclusions in a clear form in the revised versions.

Line 14: “the Dark-flies returned back to the light” Does this refer to DF65G flies?

Line 36: “environment to see evolution” Maybe: “… to observe evolution...”

Line 54: “between extent species” Maybe: “Between several species”?

Line 64: “...or alternative...” Do the authors mean “consecutive”? This paragraph should be re-written for clarity. In particular lines 66-68 are hard to comprehend by this reviewer.

Lines 82-88: Another control for DF65G flies should be Dark-flies treated exactly the same as the experimental group but without light. If this is what was done, authors should clarify. It would be nice to observe any dosage effect between flies raised in light for various numbers of generations.

There are two possible mechanisms for the sensory trade-off that the authors observe. One is a per-existing stimulus-dependent plasticity, whereby brain development of these sensory areas is a function of sensory input (activity-dependent plasticity). The other mechanism for the trade-off would be “hard-wired” changes of the sensory areas resulting from evolutionary selection. If the former mechanism is the case, we should expect to observe the differences in sensory areas between Dark-flies and DF1G, while in the case of the latter mechanism, the evolutionary selection, the differences should only be observed between Dark-flies (or DF1G flies) and the DF65G flies. The authors should clarify their motivation for setting the control groups.

Line 98: the line should read: “...12:12 LD lighting conditions...”

Lines 194-198: authors should state the N (number of flies) for each group across the comparisons.

Line 202: the text is probably referring to Fig. 2D, not Fig 3D .

Lines 208-214: Should the text be referring to Fig. 3 rather than Fig. 4? If so, then it makes sense. Otherwise it is difficult to see how these results are reflected in the figure.

Lines 224-232: the text is probably referring to Fig. 4, not Fig 5. The authors state that there was no effect on OL size in Fig. 4 yet there seems to be a significant difference between the Dark-flies and the other three categories in terms of OL size.

Line 235 (and elsewhere): should “main effect” be stated as “major effect”?

Lines 258-260: If the difference in OL and AL sizes were significant between Dark-flies and DF1G flies, wouldn’t this suggest a developmental rather than evolutionary cause? Are the authors suggesting that a combination of developmental and evolutionary mechanisms is implicated in the observed differences? Please clarify.

Line 295: “… i.e. mechanosensory system ...” “e.g.”?

Lines 292-304: this paragraph is difficult understand and the references to the figure and the table are unclear. Should be re-written.

Line 380: “the light of darkness” should read “the light or darkness”

Figure 1: The number of flies in each group should be stated in the figure.

Figure 2: Label the fly groups in the figure.

Figure 3: Add the number of flies in each category. Add p-values or other indicators of significance to the figure. Confidence intervals are hard to read for DF65G group for (OL and AL).

Figure 4: Add the legend. Units on y-axis are not defined. Confidence intervals are hard to read for DF651 group for (OL and AL).
